# Structural Refinement by Direct Mapping Reveals Assembly Inconsistencies near Hi-C Junctions

**DOI:** 10.3390/plants12020320

**Published:** 2023-01-10

**Authors:** Luca Marcolungo, Leonardo Vincenzi, Matteo Ballottari, Michela Cecchin, Emanuela Cosentino, Thomas Mignani, Antonina Limongi, Irene Ferraris, Matteo Orlandi, Marzia Rossato, Massimo Delledonne

**Affiliations:** 1 Department of Biotechnology, University of Verona, Strada Le Grazie 15, 37134 Verona, Italy; 2 Genartis srl, Via IV Novembre 24, 37126 Verona, Italy

**Keywords:** de novo genome assembly, optical mapping, Hi-C, assembly refinement

## Abstract

High-throughput chromosome conformation capture (Hi-C) is widely used for scaffolding in de novo assembly because it produces highly contiguous genomes, but its indirect statistical approach can introduce connection errors. We employed optical mapping (Bionano Genomics) as an orthogonal scaffolding technology to assess the structural solidity of Hi-C reconstructed scaffolds. Optical maps were used to assess the correctness of five de novo genome assemblies based on long-read sequencing for contig generation and Hi-C for scaffolding. Hundreds of inconsistencies were found between the reconstructions generated using the Hi-C and optical mapping approaches. Manual inspection, exploiting raw long-read sequencing data and optical maps, confirmed that several of these conflicts were derived from Hi-C joining errors. Such misjoins were widespread, involved the connection of both small and large contigs, and even overlapped annotated genes. We conclude that the integration of optical mapping data after, not before, Hi-C-based scaffolding, improves the quality of the assembly and limits reconstruction errors by highlighting misjoins that can then be subjected to further investigation.

## 1. Introduction

Accurate genome assemblies are required for robust downstream genomic analysis, including applications such as variant calling, differential expression profiling and pangenome analysis. Several approaches have been developed and benchmarked to verify and improve the accuracy of de novo genome assemblies at the nucleotide level [1,2,3,4]. However, structural accuracy is equally important, but it is much more laborious and complex to validate.

Advances in genome sequencing technologies and assembly algorithms currently allow the reconstruction of the full, gapless sequence of entire chromosomes [5] and even entire genomes [6,7], as demonstrated by the recent completion of the full human genome [8]. Most workflows rely on long-read technologies to generate contigs, but even the longest reads are usually insufficient to reconstruct full chromosomes. To achieve chromosome-scale assemblies, contigs must be connected by scaffolding technologies–principally high-throughput chromosome conformation capture (Hi-C) and/or optical genome mapping.

Hi-C captures genome-wide chromatin interactions in the nucleus and quantifies the interaction frequency of different loci. It involves the preparation of a chromosome conformation capture library followed by short-read sequencing. Given the assumption that intra-chromosomal interactions are more frequent than inter-chromosomal ones and that intra-chromosomal interaction frequency is directly related to the two-dimensional distance along the DNA sequence, Hi-C can statistically determine the physical proximity between genomic regions. This can be exploited for genome scaffolding by ordering and orienting contigs according to the likelihood that two contigs are in physical proximity on the same chromosome. Because intra-chromosomal interactions can span the entire chromosome, this technique enables genome reconstruction at the chromosome level [9]. In contrast, optical mapping involves the direct imaging of DNA molecules. Ultra-high-molecular-weight (UHMW) DNA is labelled at nicking-enzyme recognition sites, and long stretches (>100 kb) of DNA flowing through nanochannels are then scanned and digitalised, recording the position of labels in the DNA sequence. Such molecules can be assembled using an overlap layout consensus algorithm to generate a physical map suitable for the alignment of contigs. This procedure, known as hybrid scaffolding, usually requires contigs >100 kb derived from next-generation sequencing (NGS) data and involves two steps: the identification and correction of incongruences between maps and NGS sequences followed by the anchoring of corrected sequences to the maps, thus generating scaffolds.

In de novo genome assembly workflows, Hi-C is either used alone or applied to existing hybrid scaffolds because it can capture more distant interactions, enabling the reconstruction of whole chromosomes [10]. For example, the Vertebrate Genome Project applies Hi-C to existing optical maps, whereas the European Reference Genome Atlas relies solely on Hi-C scaffolding technology [11]. Hi-C also benefits from more straightforward laboratory protocols than optical mapping, and several companies already distribute commercial Hi-C kits. In contrast, optical mapping is dependent on laborious sample preparation and handling procedures, and it is not always possible to extract the required UHMW DNA. Furthermore, Hi-C libraries can be analysed using standard short-read sequencers, whereas a dedicated instrument is required to acquire optical maps.

Despite the practical advantages of Hi-C, its probabilistic approach can lead to scaffolding errors such as contig misplacement and misorientation [12,13,14,15,16], especially with short contigs and scaffolds [10,12]. For example, Hi-C scaffolding led to 21 misplacement and misorientation errors (affecting 83 scaffolds) in the goat (*Capra hircus*) genome assembly [15], as well as hundreds of such errors in chromosome-scale de novo assemblies of the human, mouse and *Drosophila melanogaster* genomes [14]. The use of Hi-C for scaffolding without manual revision, therefore, creates a demand for de novo assembly validation. Because optical mapping is an orthogonal technique that relies on direct DNA imaging, it has also been used to detect and correct structural errors within NGS-derived genome assemblies [17,18]. For example, optical mapping improved the structural accuracy of the mouse reference genome and a complex region in the human genome (1q21.1–q21) that was integrated into the GRCh38 reference assembly [19]. However, despite its advantages, few studies have used optical mapping data as a validation tool of the assembly [20,21,22,23], and optical maps have not been used thus far to validate or correct Hi-C-based scaffolds.

Here we used optical mapping to assess Hi-C de novo genome reconstructions. Initially, we applied the optical mapping approach to an in-house genome assembly before extending it to four other publicly available genomes. Validation with optical maps identified and corrected many Hi-C scaffolding errors suggesting a manual revision of conflicting regions, some of which comprise coding sequences. These results will serve as a guideline for genome assembly projects and possibly for the revision of completed genomes in order to maximise the accuracy of structural genome reconstructions.

## 2. Results

### 2.1. De Novo Assembly and Structural Validation of the HAEMATOCOCCUS Lacustris Genome

We generated long-read sequences of *H. lacustris* using Oxford nanopore Technology (ONT), producing 12 Gb of data comprising two million reads with an N50 value of 15 kb (Table S1). The reads were assembled de novo using Flye, yielding an assembly of 272 Mb with an N50 value of 250 kb (Table S2). The ONT assembly was polished using ONT, and Illumina reads to refine residual sequence errors. BUSCO analysis identified 1476 complete genes, indicating good completeness (97.1%) but a high duplication frequency of 72.7%. A subsequent purging step reduced the BUSCO duplication frequency to 10.6% while maintaining high completeness (92.6%). The assembly size was reduced to 150 Mbp (Table 1). A comprehensive description of *the H. lacustris* genome is being reported elsewhere [24].

An initial scaffolding step was carried out by Hi-C to order and orient contigs. A Hi-C library prepared from *H. lacustris* nuclei was sequenced using Illumina and generating 90 Gb of sequence data. This was integrated with the long-read data to generate a highly contiguous, chromosome-scale assembly. Specifically, the scaffolding process joined 930 contigs of the ONT assembly (91% of the assembled sequence) and generated a “Hi-C-scaffolded genome” comprising 32 scaffolds with an N50 value of 4 Mb (Table 1).

**Figure 1:**
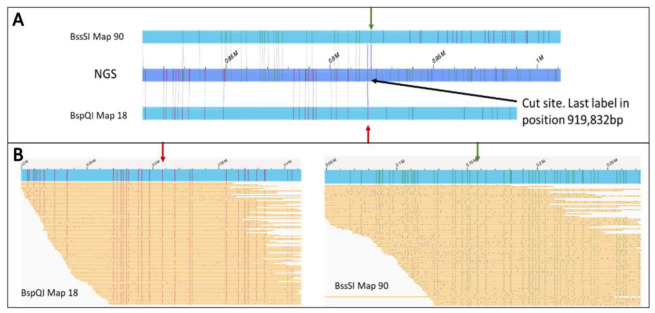
Example of inconsistency between optical maps and Hi-C-derived scaffolds in the *H. lacustris* assembly. (**A**) Alignment to the NGS assembly scaffolded with Hi-C. Red and green arrows indicate the incongruence points in the BspQI and the BssSI maps, respectively. (**B**) Raw Bionano molecules aligned on the two maps involved in the conflict.

For the structural validation of the *H. lacustris* genome assembly based on the complete ONT dataset and the Hi-C scaffold, we produced two independent optical genome maps using the enzymes BspQI and BssSI, generating 198 and 163 Gb of sequencing data, respectively. The sizes of the resulting assembled genome maps were 323 and 370 Mb, with N50 values of 520 and 630 kb, respectively. The size of the reconstructed genome maps highlights how both haplotypes were fully reconstructed using optical maps. The hybrid scaffolding generated a genome assembly of 175 Mb with a scaffold N50 of 655 kb. This revealed a large number of inconsistencies between the Hi-C scaffold and optical maps, which fragmented the Hi-C scaffolded genome and reduced the N50 value (Table 1). A total of 864 incongruences were found, 455 of which were located near the 296 gaps introduced by Hi-C and classified as scaffolding errors by manual inspection. The number of conflicts solely arising from Hi-C incorrect gap size estimation was 14. Figure 1A shows an incongruence in which optical maps and NGS scaffolds (shown as in silico digested maps) align correctly until the mismatch point. Downstream, the labelling differs between the two datasets, highlighting a genomic region with a different structure in the two maps. The correctness of optical map reconstruction was confirmed by showing that two independent maps based on different enzymes uncovered the same incongruence. Furthermore, numerous Bionano molecules supported the reconstruction at the conflicting region and confirmed the incorrect assembly generated by Hi-C scaffolding (Figure 1B).

**Figure 2:**
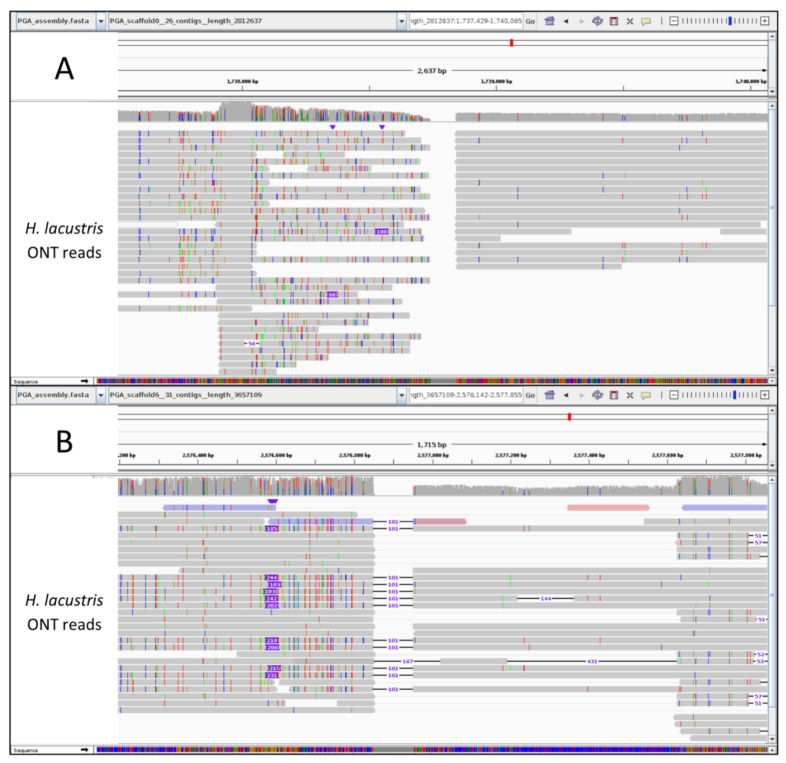
Integrative Genome Browser of two junction regions identified as potential errors by optical maps. ONT data mapped to the Hi-C-scaffolded *H. lacustris* genome show either (**A**) no reads spanning the contig junction or (**B**) reads spanning the junction but highlighting the incorrect gap size.

We then used ONT data to manually verify 62 gaps introduced by Hi-C located close to the identified conflicts. In 81% (50/62) of the revised cases (Table 2), ONT data did not support the HiC-based reconstruction because no reads spanned and properly aligned across the gap, thus confirming that Hi-C-based scaffolding introduced an error at that point (Figure 2A). In the remaining 19% of revised cases, ONT reads confirmed the contig-to-contig junction (Figure 2B). However, only a fraction of these junctions (8%) showed a good number of ONT reads in support, while <5 reads confirmed the contig connection in the remaining 11% of cases.

Hi-C-derived misjoins have been reported as more frequent for short contigs, so we evaluated the size of the contigs joined erroneously in order to establish a correlation between contig length and error probability (Table S3). Surprisingly, we found that errors did not vary significantly in relation to contig length and that the highest proportion of errors involved contigs with a consistent length of 600–650 kb. Within this range, 83% of contigs (five in every six) were involved in erroneous joins.

### 2.2. Structural Validation of Four Published Genome Assemblies

To determine whether the above findings were unique to our in-house *H. lacustris* genome assembly or could be generalised, we evaluated the presence of OM/Hi-C inconsistencies in four assemblies based on Hi-C in which the NGS-based and optical map assemblies were accessible.

**Figure 3:**
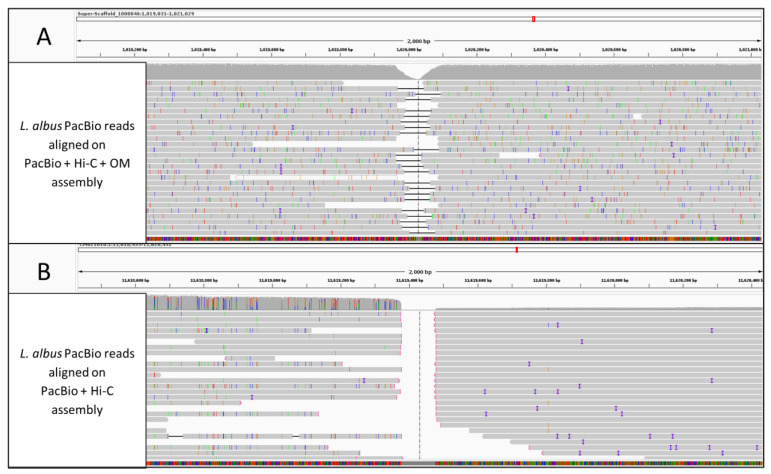
Integrative Genome Browser, visualisation of long PacBio, reads aligned on an OM/Hi-C conflict identified on the *L. albus* genome assemblies. (**A**) PacBio reads aligned on the PacBio + Hi-C + OM (after anchoring) assembly support the OM-based reconstruction. (**B**) PacBio reads aligned on the PacBio + Hi-C assembly do not support the HiC-based reconstruction.

First, we analysed two published assemblies of white lupin (*Lupinus albus* L.) cv. AMIGA. We retrieved the PacBio genome assembly scaffolded by Hi-C [25] and used the corresponding optical maps [26] to validate the assembly. This revealed 401 conflicts involving 27 scaffolds, 45 of which were located near gaps introduced by Hi-C and classified as true conflicts by manual inspection. The contiguity of the genome decreased by 50%, from 18 to 9 Mb (Table 3). The correctness of optical maps anchored scaffolds was manually verified by aligning the PacBio data to this assembly. Figure 3A shows PacBio reads properly aligned over the reconstruction obtained following the optical maps-based correction of a conflict site, supporting the final structure of the hybrid assembly and confirming the previous misplacement generated by Hi-C scaffolding (Figure 3B).

The same approach was then applied to three randomly selected genomes from the Vertebrate Genome Project: the Hawaiian crow (*Corvus hawaiiensis*), the South Island Takahe (*Porphyrio hotchstetteri*) and the American shad (*Alosa sapidissima*). All three assemblies are based on PacBio contigs anchored to optical maps before scaffolding with Hi-C data. The final genome assemblies were validated against the same optical maps by using the hybrid assembly procedure. We identified multiple conflicts between the optical maps and Hi-C scaffolds (352 for *C. hawaiiensis*, 182 for *P. hotchstetteri* and 825 for *A. sapidissima*) involving 43%, 42% and 82% of the scaffolds, respectively. Such conflicts are, therefore, widespread and can involve a large proportion of the chromosomes (Table 4). Among the identified conflicts, 5 (*C. hawaiiensis*), 11 (*P. hotchstetteri*) and 126 (*A. sapidissima*) were located near gaps introduced by Hi-C, but not due to Hi-C wrong gap-estimate and thus classified as Hi-C errors by manual inspection.

After optical maps conflict resolution, the contiguity of all three genomes decreased significantly (Table 5). The greatest impact was observed for *A. sapidissima*, where the scaffold N50 value decreased by order of magnitude from 38 to 3.6 Mb. However, following the integration of optical maps for scaffolding, the final hybrid assembly of all three genomes had a similar contiguity to the corresponding starting assembly in terms of scaffold N50 value. Moreover, the size of the gaps increased 10-fold (*C. hawaiiensis* and *A. sapidissima*) or threefold (*P. hotchstetteri*).

## 3. Discussion

Traditionally, the validation of de novo assemblies has relied on physical maps, such as those based on radiation hybrids and fluorescence in situ hybridization. More recently, innovative technologies such as optical mapping and electronic mapping have been developed to provide equivalent validation capability. We used optical maps after Hi-C scaffolding to assess the robustness of Hi-C reconstructions. We observed hundreds of incongruences in all the genome assemblies we analysed, ranging from 182 in *P. hotchstetteri* to 864 in *H. lacustris*, thus highlighting the discordance between the two reconstruction methods. Notably, a large fraction of conflicts was located near Hi-C junctions, suggesting possible misjoins reflecting the intrinsic properties of the Hi-C method. Confirming this hypothesis, we found that 83% of the putative misjoins in *H. lacustris* were also supported by ONT long reads, with similar results in the other genome assemblies. Furthermore, the correctness of optical map-based reconstruction was confirmed by aligning raw DNA molecules to the assembled maps.

As it was already reported in the literature, Hi-C is prone to generate contig misorientation and misplacement because chromosome structure is estimated statistically based on contact frequency. Several other factors also cause Hi-C errors. First, Hi-C is based on short-read sequencing that cannot uniquely map over genomic repeats, leading to low-quality alignments and the uneven distribution of genomic data. Second, restriction enzymes are used to generate the final NGS library, so performance depends on the frequency and distribution of restriction sites in the genome, potentially leading to the sparse mapping or complete omission of some contigs.

Interestingly, Hi-C errors were not restricted to low-complexity regions such as telomers or centromeres but were spread throughout most of the reconstructed chromosomes. For example, the manually identified 126 Hi-C errors in the A. sapidissima genome assembly were found in 21 out of 29 (72%) reconstructed chromosomes. Furthermore, in contrast to earlier reports suggesting that Hi-C errors are more prevalent among short contigs, we found no correlation between the error rate and contig length in the *H. lacustris* genome. The contig length with the highest frequency of errors was among the longest (600–650 kb).

The importance of optical maps as a validation technology does not only reflect the identification of scaffolding errors. Indeed, the hybrid scaffolding pipeline identifies incongruences with the NGS-based reconstructions in the first step, and the corrected sequences are anchored to the maps to generate more reliable scaffolds in the second step. Although the first step introduces “cuts” corresponding to the incongruences, integration of the maps as a scaffolding tool in the second step can restore the contiguity of the assembly. Accordingly, the N50 value of the final scaffolds may not differ considerably following the correction. This was observed in four of the five assemblies we analysed. The N50 value of the *P. Hotchstetteri* assembly was identical before and after optical maps integration. In *A. sapidissima*, which featured the greatest number of incongruences among the publicly available genome assemblies, the N50 value was only halved by hybrid scaffolding. The exception was *H. lacustris*, where the hybrid assembly significantly decreased the assembly N50 value, probably due to the high content of repeat sequences in this genome.

A second advantage provided by the optical maps hybrid scaffolding is the correct estimation of gap sizes. Optical map scaffolding is based on the measurement of physical distances between markers, whereas Hi-C cannot determine the correct gap size and introduces a user-determined fixed gap length. This was particularly evident in the *H. lacustris* assembly, where none of the gaps was correctly sized. In all the other genomes, the gap size increased up to 10-fold following the integration of optical maps. Another common limitation of NGS de novo genome assembly is tandem repeat collapsing [27], where near identical repeats are assembled as a single copy or the incorrect number of copies. This was evident in the recent telomere-to-telomere reconstruction of the human genome, where the only unresolved region was the tandem array of rRNA genes [8]. Optical maps are sequence-agnostic and can therefore provide accurate estimates for very long regions of tandem repeats [28], even those that defeat long-read sequencing.

In conclusion, in agreement with earlier studies, we demonstrated that Hi-C scaffolding could lead to contig misorientation and misidentified joints, but we suggest that these errors can be resolved by using optical maps as the final scaffolding step in order to improve the structural accuracy of genome assemblies. One possible limitation of optical mapping is its low resolution. With a density of one label every ~10 kb, only contigs longer than ~100 kb contain a sufficient amount of label sites for confident alignment [29]. A new technology from Nabsys for high-throughput electronic mapping, which is based on solid-state nanodetectors [30] and expected to provide higher resolution and accuracy than optical mapping, may overcome the limitations caused by low label density. Furthermore, current long-read technologies can easily generate de novo assemblies with contigs longer than 1 Mb [31,32,33].

## 4. Materials and Methods

### 4.1. Haematococcus lacustris Cultivation

We obtained *H. lacustris* strain K0084 from the Culture Collection of Algae at Goettingen University. Cells were seeded into flasks containing BG-11 medium and were grown photoautotrophically at 25 °C.

### 4.2. ONT Sequencing

Nuclei were isolated from 4.3 × 10^8^ *H. lacustris* cells in MEB buffer [34] (Lutz, 2011), and the nuclear DNA was extracted using the Qiagen Genomic Tip-100 (Qiagen, Hilden, Germany). After DNA quantification and quality control as above, the nuclear DNA was fragmented to ~20 kb using a g-TUBE (Covaris, Brighton, UK) and treated with a short read eliminator (Circulomics, Pacific Biosciences, Menlo Park, CA, USA) to remove short fragments [35]. A 4-µg aliquot of DNA was end-repaired and dA-tailed using the Next End Repair/dA-tailing module (New England Biolabs, Ipswich, MA, USA) and ONT libraries were prepared using the ligation protocol (SQK-LSK109) according to the manufacturer’s instructions (ONT, Oxford, UK) but with longer incubation times [36]. Approximately 15 fmol of the library was loaded into a MinION flowcell (FLO-MIN106_R9.4.1), and loading was repeated on the same flowcell after nuclease flushing (NFL_9076_v109). The sequencing run lasted ~48 h.

### 4.3. Illumina Sequencing

Whole-genome Illumina DNA-Seq libraries were prepared using the KAPA Hyper Prep Kit (Kapa Biosystems, Wilmington, MA, USA) and a PCR-free protocol. Nuclear DNA was sheared using an M220 ultra-sonicator (Covaris), adjusting the treatment time to obtain ~350-bp fragments. The size of the resulting libraries was assessed by capillary electrophoresis on a Bioanalyzer High Sensitivity DNA chip. Libraries were quantified by qPCR using a standard curve and were sequenced on an Illumina NovaSeq 6000 device to generate 119 million 150 paired-end reads (36 Gb).

### 4.4. Optical Genome Mapping

UHMW DNA was extracted from isolated *H. lacustris* nuclei using agarose plugs (Bionano Genomics, San Diego, CA, USA). After DNA quality control as above, 300 ng of DNA was labelled using 10 U/µL Nt.BspQI or 20 U/µL Nb.BssSI nicking endonuclease (New England Biolabs, Ipswich, MA, USA) with the NLRS DNA labelling kit (Bionano Genomics). Maps were acquired from nicked and labelled DNA using a Syphyr instrument (Bionano Genomics) at the ETH functional Genomic Center of the University of Zurich.

### 4.5. Hi-C Data Generation

We fixed *H. lacustris* biomass in 1% fresh formaldehyde for 20 min and then quenched it with 1.25 mM glycine. Nuclei were isolated in NIBTM buffer [37] (and Hi-C libraries were prepared using the Proximo Hi-C Plant kit v1.5 (Phase Genomics, Seattle, WA, USA) and restriction enzyme Sau3AI. The integrity and size distribution of the Hi-C library was assessed using a 2200 TapeStation (Agilent Technologies, Santa Clara, CA, USA).

### 4.6. H. Lacustris De Novo Genome Assembly

ONT raw reads were assembled using Flye v2.5 with default parameters [38]. The draft contig assembly underwent base-level refinement of residual errors using a combination of long and short reads. Briefly, ONT reads were aligned on the ONT draft assembly using minimap2 v2.17 with the-x map-ont parameter [39]. Racon v1.4.3 [40] was used for the initial polishing of long reads, followed by a second round using medaka v1.0.3 (https://github.com/nanoporetech/medaka (accessed on 20 July 2020)) and two rounds of sequence refinement using short reads in pilon v1.23 [41]. Genome completeness was assessed with BUSCO v4.0.6 [42] using chlorophyta_odb10 as a reference database. The purging and scaffolding of the polished ONT assembly using Hi-C data was carried out by Phase Genomics using the Proximo Genome Scaffolding Platform. After the automatic scaffolding procedure, the results were manually revised to correct putative errors.

### 4.7. Optical Genome Maps: Assembly and Hybrid Scaffolding

Optical genome maps obtained with two nicking enzymes (BspQI and BssSI) were independently assembled using Bionano Solve v3.6 (https://bionanogenomics.com/support/ (accessed on 10 December 2020)). Double enzyme hybrid scaffolding was generated from the Hi-C-scaffolded genome using Bionano Solve v3.6 and the hybridScaffold_two_enzymes_HiC.xml configuration file. The hybrid assemblies were imported to the Bionano Access system, and conflicts were manually verified.

### 4.8. Source of Publicly Available Sequencing Data

The *Lupinus albus* genome map was downloaded from https://www.whitelupin.fr/ (accessed on 20 December 2021) (Hufnagel B. et al.), and the Hi-C-scaffolded genome was sourced from NCBI Bioproject PRJNA592024 (Xu W. et al.). VGP assembly data for *C*. *hawaiiensis*, *P. hotchstetteri* and *A. sapidissima* were downloaded from https://genomeark.github.io/vgp-all (accessed on 20 December 2021). The NGS and map assemblies were processed by hybrid scaffolding using Bionano Solve v3.6 after modifying the nicking enzyme to match the ones used for acquisition. The hybrid assemblies were imported into the Bionano Access system, and conflicts were manually verified.

## Figures and Tables

**Table 1:** Statistical data for the *H. lacustris* assembly (ONT assembly) scaffolded with Hi-C (ONT + Hi-C) and then corrected and anchored with optical maps (ONT + Hi-C + OM).

	ONT Assembly	ONT + Hi-C	ONT + Hi-C + OM
Total assembly length	150,233,945	150,327,121	175,351,177
Total scaffold length (bp)	-	137,192,742	162,448,219
Number of scaffolds	-	32	1507
Scaffold N50 (bp)	-	4,010,071	669,654
Scaffold average length	-	4,287,273	107,796
Longest scaffold (bp)	-	9,907,970	4,400,370
Shortest scaffold (bp)	-	1,735,284	210
Number of gaps	-	930	1120
Gap size (bp)	-	93,000	25,118,148
Contigs in scaffolds	-	962	1696
Remaining contigs	1799	849	58
Remaining contig total length (bp)	150,233,945	13,134,379	12,902,958
Remaining contig N50 (bp)	230,876	49,528	248,743
Remaining contig average length (bp)	83,510	15,470	222,465
Remaining contig maximum length (bp)	987,270	438,472	925,569
Remaining contig minimum length (bp)	501	501	80,956

**Table 2:** Classification of junction errors based on manual evaluation of the alignment of ONT reads on the Hi-C-scaffolded *H. lacustris* genome assembly. The table shows the number and percentage of errors identified by alignment to optical maps and confirmed by manual inspection of ONT read mapping at the conflict site.

Type	Number	Percentage
Confirmed error	50/62	81%
Non-confirmed error	12/62	19%

**Table 3:** Statistical data for the *L. albus* assembly generated with PacBio data (PacBio assembly [25]), the correction with optical maps (PacBio + Hi-C after OM conflict resolution) and the final hybrid scaffold integrating the PacBio assembly with Hi-C and optical maps scaffolding (PacBio + Hi-C + OM).

	PacBio + Hi-C	PacBio + Hi-C After OM Conflict Resolution	PacBio + Hi-C + OM
Total assembly length (bp)	558,896,430	558,896,430	566,549,843
Total scaffolds length (bp)	500,601,628	494,945,822	506,701,445
Number of scaffolds	67	169	124
Scaffold N50 (bp)	18,661,206	9,318,959	11,119,386
Scaffold average length (bp)	7,471,666.09	2,928,673.50	4,086,301.98
Longest scaffold (bp)	25,248,489	19,026,298	22,043,127
Shortest scaffold (bp)	11,445	4439	4439
Number of gaps	1764	1764	1823
Gap size (bp)	1,532,853	1,532,853	9,186,266
Contigs in scaffolds	1831	1933	1947
Remaining contigs	1513	1812	1798
Remaining contig total length (bp)	58,294,802	63,950,608	59,848,398

**Table 4:** Classification of conflicts between optical maps and Hi-C scaffolds based on the manual evaluation of incongruent regions. The table shows the total number of conflicts identified by alignment to optical maps and the number of affected scaffolds compared to the total number of chromosomes.

	*Corvus hawaiiensis*	*Porphyrio hotchstetteri*	*Alosa sapidissima*
Number of conflicts	352	182	825
Number of scaffolds cut/Number of chromosomes	21/48	30/71	24/29

**Table 5:** Statistical data for the Corvus hawaiiensis, Porphyrio hotchstetteri and Alosa sapidissima scaffolded assemblies generated from PacBio data in the Vertebrate Genome Project (Hi-C), the assembly statistics after conflicts resolution (After OM conflict resolution) and the final hybrid scaffold integrating the PacBio assembly with Hi-C and optical maps scaffolding (OM.).

	*Corvus hawaiiensis*			*Porphyrio hotchstetteri*			*Alosa sapidissima*		
	Hi-C	After OM Conflict Resolution	OM	Hi-C	After OM Conflict Resolution	OM	Hi-C	After OM Conflict Resolution	OM
Total assembly length (bp)	1,151,594,481	1,151,593,281	1,169,606,052	1,270,322,674	1,270,322,434	1,280,192,390	903,564,947	903,564,347	951,311,491
Total scaffold length (bp)	1,089,055,265	997,131,828	1,136,185,454	1,221,472,023	1,167,038,074	1,229,274,670	898,428,452	782,348,280	924,915,787
Number of scaffolds	48	69	65	71	123	107	29	378	147
Scaffold N50 (bp)	76,278,832	35,694,304	65,807,562	71,566,193	43,238,439	71,566,193	38,440,066	3,604,007	35,651,408
Scaffold average length (bp)	22,688,651.35	14,451,185.91	17,479,776.22	17,203,831.31	9,488,114.42	11,488,548.32	30,980,291.45	2,069,704.44	6,291,944.13
Longest scaffold (bp)	123,451,405	105,815,375	120,194,775	224,114,340	105,985,913	170,159,902	56,504,578	18,980,070	62,312,241
Shortest scaffold (bp)	86,660	17,826	17,826	25,768	2832	2832	22,192	1287	1287
Number of gaps	294	294	384	327	327	406	1639	1641	1983
Gap size (bp)	1,813,227	1,813,227	19,825,998	5,316,249	5,316,249	15,186,205	4,995,812	4,995,812	52,742,956
Contigs in scaffolds	342	363	449	398	442	506	1668	1993	2108
Remaining contigs	139	472	386	103	242	178	44	522	407
Remaining contig total length (bp)	62,539,216	154,461,453	33,420,598	48,850,651	103,284,360	50,917,720	5,136,495	121,216,067	26,395,704
Remaining contig N50 (bp)	16,938,386	9,525,645	551,250	12,827,660	16,562,103	12,827,660	233,926	1,091,412	130,353
remaining contig average length (bp)	449,922.42	327,248.84	86,581.86	474,278.17	426,794.88	286,054.61	116,738.52	232,214.69	64,854.31
Longest remaining contig (bp)	23,235,100	33,421,925	7,094,517	19,484,417	20,414,059	19,484,417	403,292	6,672,834	1,756,549
Shortest remaining contig (bp)	2297	641	641	3912	504	504	2376	659	659

## Data Availability

Raw sequencing and mapping data presented in this work are openly available at NCBI database (Bioproject accession number PRJNA904687).

## Supplementary Materials

The following supporting information can be downloaded at: https://www.mdpi.com/article/10.3390/plants12020320/s1, Table S1: Statistical analysis of the Haematococcus *lacustris* sequencing and mapping data; Table S2: Statistical analysis of the Haematococcus *lacustris* draft genome assembly data; Table S3: Number of contigs in Hi-C scaffolds grouped by contig length and relative percentage of erroneous joins.
